# Ileo-sigmoid knotting: a review of 61 cases in Kenya

**DOI:** 10.11604/pamj.2016.23.198.6255

**Published:** 2016-04-15

**Authors:** Philip Blasto Ooko, Seno Saruni, Mark Oloo, Hillary Mariko Topazian, Russell White

**Affiliations:** 1 Department of Surgery, Tenwek Hospital, Bomet, Kenya

**Keywords:** Ileosigmoid knotting, intestinal obstruction, volvulus, treatment, outcome

## Abstract

**Introduction:**

Ileo-sigmoid knotting (ISK) is a rare cause of bowel obstruction in which the ileum twists around the sigmoid colon. It is associated with rapid bowel gangrene and a high mortality rate. Little has been published about this condition in Kenya. The objective was to determine the presentation, management, and outcome of patients with ISK.

**Methods:**

A seven year (January 2008-December 2014) retrospective chart review of patients managed for ISK at Tenwek Hospital in Bomet, Kenya.

**Results:**

A total of 61 cases were identified, with a mean age of 35.8 years (range 2-68), and mean symptom duration of 1.6 days (range 3 hours-7 days). Gangrene was noted to involve both the ileum and colon in 45 patients, the ileum only in 9 patients, and the sigmoid colon only in one. Resection and primary anastomosis was carried out in most cases of gangrenous ileum (48/54, 89%) and gangrenous sigmoid colon (34/46, 74%), while resection and stoma was performed in 8 patients with gangrenous colon. Death occurred in 7 (11.5%) patients due to severe sepsis and multisystem organ failure. Morbidities were noted in 15 (24.6%) patients, including surgical site infection (8, 13.1%), respiratory insufficiency (4, 6.6%), fascial dehiscence (3, 4.9%) and anastomotic leak (2, 3.2%). The mean duration of hospitalization was 8.3 days (range 1-26).

**Conclusion:**

In this review, though retrospective in nature, ISK was noted to have high rates of bowel gangrene. In the appropriate setting, resection and primary anastomosis can be safely carried out in most cases of gangrenous colon.

## Introduction

Ileo-sigmoid knotting (ISK) is a rare cause of acute intestinal obstruction [1–3] that occurs when a loop of ileum wraps around the base of a redundant sigmoid colon [3–5]. This condition is associated with high rates of gangrene involving the ileum, sigmoid colon, and at times, even extending to the caecum and ascending colon [3, 5]. Anatomical factors postulated to predispose to the development of ISK include: a long small bowel mesentery and freely mobile small bowel, and a redundant sigmoid colon on a narrow mesenteric base [2, 5]. Though early surgery is recommended, as ISK is associated with early bowel gangrene and a high mortality rate, most cases are diagnosed intra-operatively due to the rarity of this condition and nonspecific clinical, laboratory, and X-ray features [1, 3]. Use of Computerized tomography (CT) has led to an increased preoperative diagnostic accuracy, but is most useful when diagnosis is in doubt [6, 7]. The purpose of this study was to review the presentation, management, and outcome of patients with ISK.

## Methods

A 7 year retrospective chart review of patients managed for ISK at Tenwek Hospital in Bomet, Kenya from January 1, 2008 to December 31, 2014 was done. Cases were defined as patients with ISK confirmed at laparotomy. Data concerning patient demographics, presenting signs and symptoms, laboratory and imaging findings, management, complications, outcome and duration of hospitalization were recorded from the individual case records. The significance of differences was assessed using Fisher's exact test. P-values less than or equal to 0.05 were accepted as significant. All patients presenting with features of intestinal obstruction (IO) received appropriate intravenous fluid resuscitation, empiric antibiotics especially when presence of gangrenous bowel was suspected, and correction of electrolyte and acid-base derangements where present. The diagnosis of these patients with IO was mainly based on clinical, and plain X-ray findings, with the subsequent management based on the preoperative diagnosis, patients overall status and intraoperative findings. All laparotomies were carried out by a consultant or senior resident under close supervision by a consultant.

## Results

**Figure 1:**
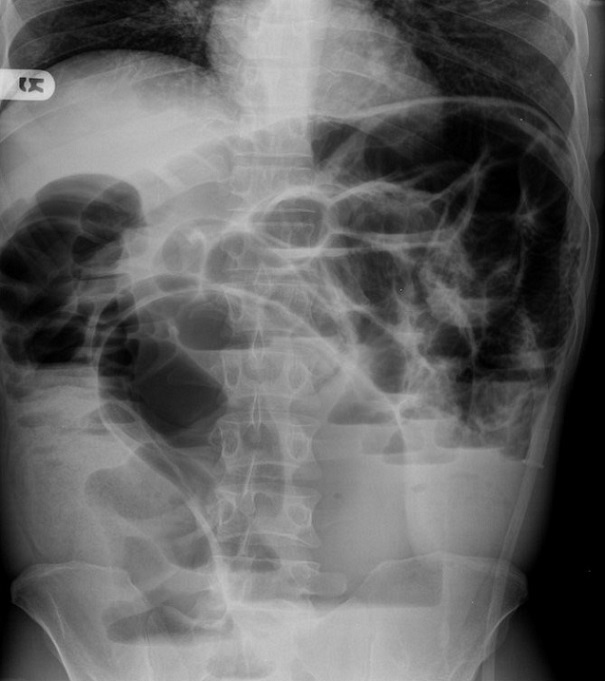
Plain upright abdominal X-ray showing dilated sigmoid colon and dilated small bowel with multiple air-fluid levels

**Figure 2:**
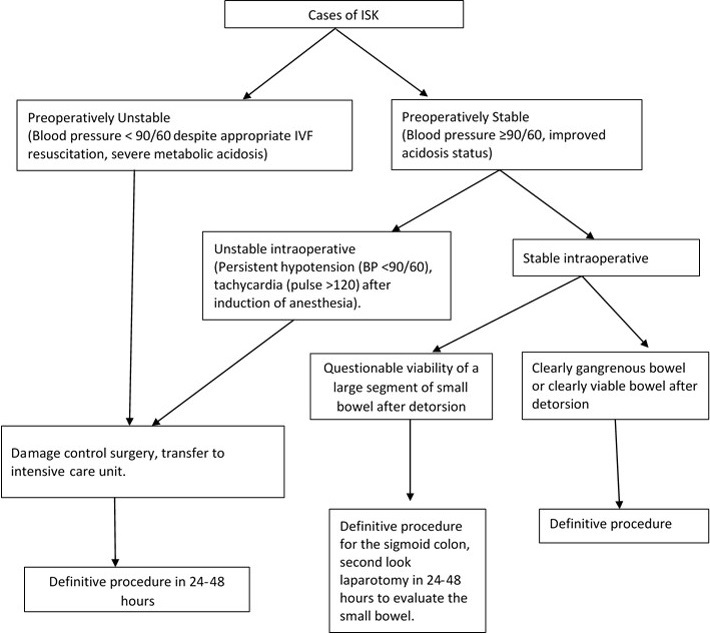
Management algorithm of cases of ISK

Sixty one cases of ISK were noted, representing 38% of the 159 cases of sigmoid volvulus seen during the study period. The group was comprised of 51 (83.6%) males and 10 (16.4%) females and had a mean age of 35.8 years (range 2-68). Peak incidence of ISK was noted in the 31-40 years age group, and the majority of cases (70.5%) between 11-50 years of age (Table 1). Patients presented after a mean duration of 1.6 days (range 3 hours-7 days) after symptom onset, with the majority (39, 63.9%) presenting within 1 day or less. The most common signs and symptoms were abdominal pain (98.4%), abdominal tenderness (90.1%), abdominal distension (83.6%), vomiting (78.4%), constipation (75.4%), and peritonitis (45.6%). Shock, defined by systolic blood pressure below 90 mm Hg, was noted in 10 patients. Leukocytosis, as defined by a white blood cell count above 12,000 per mm3 was noted in 35 (57.4%) cases. A plain upright abdominal X-ray was obtained in all cases, but the findings documented in 52 (85.2%) cases. Main findings included small bowel distension with multiple air fluid levels (32, 61.5%), and large bowel distension (25, 48.1%) (Figure 1). Rigid sigmoidoscopy was performed in four patients who presented with features of IO, no peritonitis on examination and X-ray findings suggestive of sigmoid volvulus. Detorsion was unsuccessful in one patient who had an emergency laparotomy, and inconclusive in three patients who subsequently underwent laparotomy after worsening of clinical findings over the course of 4-16 hours of observation. One patient had ISK diagnosed via a CT scan after presenting with features of IO, minimal tenderness on physical exam, but with free fluid in the pelvis noted on ultrasound. ISK was confirmed in all cases at laparotomy, with the preoperative diagnostic accuracy noted at 26.2%. The number of cases with gangrene was higher in the ileum (54, 88.5%) than in the sigmoid colon (46, 75.4%) but this did not reach statistical significance (p = 0.1). However, bowel gangrene was noted to extend beyond the sigmoid colon to involve the mid transverse colon in one case, and the caecum in two cases (Table 2). The incidence of gangrene in those presenting within 24 hours and those presenting after 24 hours of symptom onset was similar ( 34, 87.2% vs. 20, 90.9%, P = 1). The operative procedures undertaken were determined mainly by the patients overall physiological status and presence or absence of gangrene at laparotomy (Figure 2). Resection and anastomosis were carried out in most cases of gangrenous ileum (48/54, 88.8%) and gangrenous sigmoid colon (34/46, 73.9%). Definitive procedure was undertaken at the initial laparotomy in 36 (59%) cases, at the second look in 21 (34.4%) cases, while 4 (6.6%) cases died before the planned second look laparotomy. Morbidities were noted in 15 (24.6%) patients, including surgical site infection in 8 (13.1%), respiratory failure in 4 (6.6%), fascial dehiscence in 3 (4.9%), enterocutaneus fistula in 2 (3.2%), and renal failure one (1.6%). At discharge, a total of 7 (11.5%) mortalities were noted. Deaths occurred only in patients with gangrenous bowel, from severe sepsis and multi-system organ failure. Four patients who presented in septic shock, unresponsive to fluid resuscitation, had resection of the gangrenous bowel, with both ends closed with suture or staplers, peritoneal lavage and closure of the skin, with fascia left open. They were then transferred to the intensive care unit for continued resuscitation, with a planned second look laparotomy in 24-48 hours, but they succumbed within 2-24 hours after the initial procedure. The other 3 patients died within 4-17 days after admission, one of with an anastomotic leak after double resection and anastomosis. Of the patients who successfully underwent a planned second look laparotomy (n = 21) after resection of the gangrenous bowel, most had a double anastomosis, with only two cases having a colostomy and ileo-ileal anastomosis. The second look laparotomy was due to persistent intraoperative hypotension (7, 33.3%), to assess small bowel viability (6, 28.6%) and unclear or not indicated in seven cases. Significant delays from admission to operation were noted in 9 patients who presented with minimal tenderness, no guarding or rigidity, blood pressure readings within normal range and nonspecific findings on X-ray, in whom a diagnosis of acute abdominal emergency was not made. A notable example is a 45 year old male patient who presented with 6 days history of abdominal pain, vomiting and constipation, with findings of epigastric tenderness on examination, vital signs within normal and elevated amylase at four times normal range, who was managed as acute pancreatitis, only to deteriorate within 48 hours of admission. At laparotomy, he was found to have necrotic ileum and sigmoid colon, and died within 24 hours after damage control surgery. The mean length of hospitalization was 8.3 days (range 1-26) with 49% being admitted for a period of 7-9 days.

**Table 1:** Age and sex distribution of cases with ISK by decade (n = 61)

Age (years)	Male	Female	Total (percentage)
0-10	3	1	4 (6.6%)
11-20	8	1	9 (14.8%)
21-30	8	3	11 (18%)
**31-40**	**10**	**4**	**14 (23%)**
41-50	8	1	9 (14.8%)
51-60	8	0	8 (13.1%)
61-70	6	0	6 (9.8%)

**Table 2:** The number of cases grouped by bowel status and type of procedure employed (n = 61)

Laparotomy finding	Procedure	No. of patients	Deaths
Viable ileum and viable sigmoid colon (n = 6)	Ileal detorsion, sigmoid resection and primary anastomosis	5	0
	Ileal detorsion, sigmoid detorsion	1	0
Viable ileum and gangrenous sigmoid colon (n = 1)	Ileal detorsion, sigmoid resection and primary anastomosis	1	0
Gangrenous ileum and viable sigmoid colon (n = 9)	Ileal resection and primary anastomosis, sigmoid resection and primary anastomosis	8	0
	Ileal resection and primary anastomosis, sigmoid resection and Hartman's procedure	1	0
Gangrenous ileum and gangrenous sigmoid colon (n = 42)	Ileal resection and primary anastomosis, sigmoid resection and primary anastomosis	32	2
	Ileal resection and primary anastomosis, sigmoid resection and Hartman's procedure	7	1
	Ileal resection, sigmoid resection	2	2
	Ileal resection and ileostomy, sigmoid resection and primary anastomosis	1	0
Gangrenous sigmoid colon, transverse colon and gangrenous ileum (n = 1)	Ileal resection, Total abdominal colectomy and ileorectal anastomosis	1	0
Gangrenous sigmoid colon, transverse colon, descending colon, caecum and gangrenous ileum (n = 2)	Ileal resection, total abdominal colectomy	2	2

## Discussion

ISK is an important cause of intestinal obstruction (IO), with a varied geographical distribution. It is a common cause of IO in certain African, Asian and Middle Eastern countries, where it may account for up to half of all cases of sigmoid volvulus (SV) [3, 5, 8, 9]. The ratio of ISK/SV was 38% in this study. ISK has a propensity for development of intestinal gangrene, reported at 73-94% [5, 9–11], either involving the ileum and/or sigmoid colon, and at times, extending beyond the bowel components making the knot [2, 5, 10]. Thus early diagnosis and operative treatment are vital in the reversal of the effects of endotoxemia from gangrenous bowel and the prevention of vascular compromise in those with viable bowel at presentation [3, 5, 10, 12]. This condition has been reported to have a male predominance [2, 3, 13], peak incidence at 30-45 years [3, 8], and a mean age of 40-49 years [2, 5, 8, 9, 13]. Similar findings were noted in this series except for the slightly lower mean age of 35.8 years. Most patients present within 2-4 days of symptom onset, with the main complaints being abdominal pain (100%), constipation (98-100%), abdominal distension (94-96%), and vomiting (84-89%) [5, 8–11, 14]. Peritonitis has been noted in 37-69% of cases [5, 10, 11, 14]. The most common features noted on X-ray include distended loops of small bowel with multiple air fluid levels and distended colon [10–13]. Some X-rays may show apparent SV only, free air due to perforation or may be normal [9, 11]. Due to the nonspecific nature of the presentation, findings and plain X-ray features, the preoperative diagnostic accuracy has been reported to 0-25% [8, 9, 15]. In this series, the symptoms, signs, X-ray features, and preoperative diagnostic accuracy were similar to those of other studies. A clinical picture of small bowel obstruction, radiographic evidence of large bowel obstruction and inability to insert an endoscope have been proposed as a useful diagnostic triad of ISK [12, 16]. Use of a rigid sigmoidoscope in this series may have contributed to an inconclusive diagnosis in the three patients due to inability to reach the point of torsion. It is also important to note that this triad was applicable in a small proportion of our patients who did not have peritonitis or in septic shock. Some authors argue that a preoperative diagnosis of ISK is not important, but paramount is to operate as soon as a reasonable diagnosis is made [9]. This may be readily applicable in patients presenting with symptoms of IO and peritonitis, septic shock, or those with a pneumoperitonium on X-ray. Patients with ISK who present with minimal tenderness and nonspecific features on plain X-ray and laboratory findings within the normal range, may have significant diagnostic delay, as was noted in 9 patients in this series. A CT scan may be useful in this subset of patients [7, 12].

Management should begin at presentation with aggressive fluid resuscitation and correction of electrolyte and acid-base imbalances where present [2, 7, 12, 13]. Empiric antibiotic therapy should be commenced early due to the high incidence of bowel gangrene and associated bacterial translocation, and continued after the operation especially in gangrenous bowel [2, 7, 13]. The operative procedure undertaken should be based on the degree of physiological disturbance, operative risk assessment and intraoperative findings [2, 5, 12]. Patients who are unstable as defined by a blood pressure <90/60 mm Hg and a pulse >120 beats/min, either preoperatively, or after the induction of anesthesia, despite appropriate fluid resuscitation should have damage control laparotomy as the default procedure. This involves enblock resection of the volvularized segment with care not to spill bowel contents, closure of the bowel edges with suture or staples when available, peritoneal lavage and temporary abdominal closure [6]. A planned second look laparotomy should be performed 24 - 48 hours later based on the patient's response to resuscitation. The four patients who died within 2-24 hours after damage control surgery illustrate the significant degree of physiological disturbance that can occur in this condition. Patients who are stable intra-operatively, may have a definitive procedure done at the initial laparotomy. If the segment of small bowel involved in the knot is large and of questionable viability with risk of short gut syndrome if resected, then a second look laparotomy may be justifiable to assess viability after 24 - 48 hours. Detortion of the knot is controversial. It is recommended if the knots are viable or if the viability of the segments is questionable [5, 12, 16]. In presence of frankly gangrenous bowel or septic shock, enblock resection is advised due to risk of significant increase in the duration of procedure, rupture of the distended bowel segments, and/or release of toxins from the untwisted segment of bowel [2, 5, 12]. In this series, detortion was performed in clearly viable bowel, while in gangrenous cases, the sigmoid was resected first, allowing the ileum to be easily detorsed and the nonviable edges resected. This was to spare as much small bowel as possible to reduce the probability of having short gut syndrome [5]. Traditionally, resection with colostomy has been advocated for management of gangrenous sigmoid due to poor vascularity and risk of anastomotic leak [7, 13, 16].

Newer studies have indicated that primary anastomosis in gangrenous sigmoid colon can be safely carried out without significant complications [2, 5, 12]. A resection and anastomosis (RA) was carried out successfully in 74% of the patients with gangrenous sigmoid volvulus in this series with an anastomotic leak noted in only one patient (2.9%, n = 34). Patients had a RA of gangrenous sigmoid in the initial or at the second look laparotomy if they were healthy, had no gross contamination, and the bowel edges looked viable, with a rich blood supply, and a tension free anastomosis could be achieved. However, a stoma should be used in cases where there is limited or little expertise, the viability of the stump is doubtful, and there is a significant difference in diameter of the bowel edges [9, 10, 14]. Resection and primary anastomosis of a viable sigmoid has also been advocated due to risk of recurrence of the volvulus by the redundant loop [2, 5, 12]. Resection is usually limited to the redundant and freely mobile segment of the sigmoid colon that has cause the torsion except in cases of megacolon [5]. This was carried out in most cases in this series with viable sigmoid colon. Management for the ileum includes operative detortion if viable and resection with primary anastomosis if gangrenous [2, 6, 12]. The mortality rate for ISK has been reported to be as variable as 16-44% [1, 2, 5, 9–11, 14, 17]. The slightly lower mortality rate may have been secondary to the use of critical care adjuncts in this series. Older age (>60 years), presence of gangrenous bowel, and delayed presentation have been noted as factors related to higher mortality [5, 10].

## Conclusion

ISK is a rare cause of bowel obstruction, accompanied by high rate of bowel gangrene. A high index of suspicion, prompt and effective surgery, and appropriate post-operative care are vital in the management of this condition. Resection and primary anastomosis can be safely carried out in most cases of gangrenous ISK.

### What is known about this topic

- Ileo-sigmoid knotting (ISK) is a rare cause of bowel obstruction associated with high rates of bowel gangrene;
- Features of small bowel obstruction, radiographic evidence of large bowel obstruction and inability to insert an endoscope may be a useful diagnostic triad of ISK;
- Resection with colostomy has been advocated for management of gangrenous sigmoid due to poor vascularity and risk of anastomotic leak.

### What this study adds

- Patients with ISK, who present with minimal tenderness and non-alarming features on plain X-rays and blood work, may have significant diagnostic delay;
- Patients who remain hypotensive despite appropriate resuscitation should have damage control laparotomy as the default procedure;
- In the appropriate setting, resection and primary anastomosis can be safely carried out in most cases of gangrenous colon.
